# Frailty Syndrome and Oxidative Stress as Possible Links Between Age-Related Hearing Loss and Alzheimer’s Disease

**DOI:** 10.3389/fnins.2021.816300

**Published:** 2022-01-18

**Authors:** Juan Carlos Alvarado, Verónica Fuentes-Santamaría, José M. Juiz

**Affiliations:** Facultad de Medicina, Instituto de Investigación en Discapacidades Neurológicas (IDINE), Universidad de Castilla-La Mancha, Albacete, Spain

**Keywords:** aging, dementia, neurodegenerative, presbycusis, reactive oxygen species

## Abstract

As it is well known, a worldwide improvement in life expectancy has taken place. This has brought an increase in chronic pathologies associated with aging. Cardiovascular, musculoskeletal, psychiatric, and neurodegenerative conditions are common in elderly subjects. As far as neurodegenerative diseases are concerned dementias and particularly, Alzheimer’s disease (AD) occupy a central epidemiological position given their high prevalence and their profound negative impact on the quality of life and life expectancy. The amyloid cascade hypothesis partly explains the immediate cause of AD. However, limited therapeutical success based on this hypothesis suggests more complex remote mechanisms underlying its genesis and development. For instance, the strong association of AD with another irreversible neurodegenerative pathology, without curative treatment and complex etiology such as presbycusis, reaffirms the intricate nature of the etiopathogenesis of AD. Recently, oxidative stress and frailty syndrome have been proposed, independently, as key factors underlying the onset and/or development of AD and presbycusis. Therefore, the present review summarizes recent findings about the etiology of the above-mentioned neurodegenerative diseases, providing a critical view of the possible interplay among oxidative stress, frailty syndrome, AD and presbycusis, that may help to unravel the common mechanisms shared by both pathologies. This knowledge would help to design new possible therapeutic strategies that in turn, will improve the quality of life of these patients.

## Introduction: The Neurobiological Missing Links Between Age-Related Hearing Loss and Alzheimer’s Disease

Age-related hearing loss (ARHL) or presbycusis is a major public health burden worldwide that profoundly affects the quality of life of those who suffer from it (Mathers et al., 2000, 2008; World Health Organization [WHO], 2002, 2021). It is a progressive, chronic, and irreversible condition, which should be construed as a neurodegenerative disease, for which no curative treatment is available yet (Yamasoba et al., 2013; Melgar-Rojas et al., 2015; Parham et al., 2015; Wang and Puel, 2020). Also, it is the most frequent sensory impairment in the elderly. Estimates are that 4 out of 10 adults aged 60 years or older have some degree of limiting hearing loss (World Health Organization [WHO], 2021). Moreover, the prevalence of disabling hearing loss has been estimated at about 15% at 60 years of age, increasing up to 58% at 90 years of age, with all the consequences that this entails (World Health Organization [WHO], 2021). AD is also a progressive, chronic, and irreversible pathology without curative treatment. It represents around 70% of dementias, which affect about 55 million people worldwide (World Health Organization [WHO], 2017a; Zheng et al., 2017; Shen et al., 2018; Ralli et al., 2019; Guo et al., 2020). Life quality is severely affected since AD interferes with the ability to perform daily life activities. Therefore, autonomy and life expectancy are severely compromised (World Health Organization [WHO], 2017a; Guo et al., 2020).

Epidemiological studies show a strong independent association between ARHL and AD (Lin et al., 2011; Lin and Albert, 2014). Actually, recent data link 9% of sporadic AD to hearing loss starting at mid-life. Thus, ARHL emerges as the main preventable risk factor of AD, at least in this life period, even with causal implications (Livingston et al., 2017; Loughrey et al., 2018). Comorbidity between ARHL and AD will further aggravate the condition of the patients, multiplying health, social, economic, and sanitary impact. In sum, epidemiological data link ARHL with cognitive impairment and dementias, in particular AD, pointing to dynamic association between these two neurodegenerative conditions (Fortunato et al., 2016; Zheng et al., 2017; Shen et al., 2018; Panza et al., 2019; Ralli et al., 2019; Uchida et al., 2019; Guo et al., 2020; Llano et al., 2020; Slade et al., 2020; Johnson et al., 2021). Besides ARHL contributing to the pathogenesis of AD, the converse may also be the case. However, at present, the biological or mechanistic foundations of such interplay are unknown (Griffiths et al., 2020; Nadhimi and Llano, 2021). Several hypotheses/mechanisms have been put forth. These include existence of shared underlying pathologies, such as those of vascular origin; diminished auditory input that directly triggers brain atrophy as an expression of the complex chain of cellular events leading to dementia; overload of cognitive resources, diverted to process diminished auditory input (Uchida et al., 2019; Griffiths et al., 2020); existence of amyloid plaques (AP), intraneuronal neurofibrillary tangles (NFT) and cytoskeletal pathology in the cochlea, dorsal cochlear nucleus, superior olive, central nucleus of the inferior colliculus, medial geniculate body, primary auditory cortex and association area of the auditory cortex (Omata et al., 2016; Shen et al., 2018). These or another related hypothesis/mechanism do not exclude each other mutually. Whether such interplay is unidirectional from ARHL to AD, or bidirectional is also unknown. The challenge of testing such intricate and open-end hypotheses scenery, is the complexity and multiplicity of factors involved in the genesis and development of both neurodegenerative conditions. Frailty and related oxidative stress have recently drawn considerable attention (Kawada et al., 2015; Panza et al., 2015a,b; Huang et al., 2016; Shen et al., 2018; Wang and Puel, 2020; Misrani et al., 2021). In this review, we discuss the possibility that the oxidative stress linked to frailty, could be, at least in part, primarily involved in the interplay between ARHL and AD. We stress the need for the development and use of rodent models to target and integrate in a translational approach molecular, cellular, and behavioral mechanisms at the interphase between ARHL and AD.

## Oxidative Stress in Age-Related Hearing Loss and Alzheimer’s Disease

When the endogenous antioxidant system is overcome either by production of excess free radicals, essentially reactive oxygen and nitrogen species (ROS/RNS), limited free radical handling or both, accumulation of toxic free radicals occurs, leading to oxidative stress-induced damage to lipids and proteins in cell membranes and the cytosol, as well as to the nuclear and mitochondrial genome (Ames et al., 1993; Halliwell, 2006; Fetoni et al., 2011; Böttger and Schacht, 2013; Nimse and Pal, 2015). The deleterious effects that excess of free radicals has on cells, seemingly is critically associated with the aging process, the genesis and/or development of different neurodegenerative pathologies such as amyotrophic lateral sclerosis, Huntington, Parkinson’s and Alzheimer’s diseases and presbycusis (Ames et al., 1993; Seidman, 2000; Chauhan and Chauhan, 2006; Uttara et al., 2009; Dai et al., 2014; Huang et al., 2016).

In the auditory system, besides ARHL, oxidative stress is at the core of noise-induced hearing loss (NIHL) and drug-induced hearing loss (DIHL; Henderson et al., 2006; Le Prell et al., 2007a,b; Fetoni et al., 2013). This has led to the notion that oxidative stress is a common pathogenic pathway, shared by the above-mentioned auditory pathologies (Alvarado et al., 2015, 2018). In the peripheral auditory system, the cochlea is particularly exposed to damage from oxidative stress. This is likely due to the metabolic demands of mechanoelectrical transduction (Robles and Ruggero, 2001; Fettiplace and Kim, 2014), for which large electrochemical gradients have to be maintained, particularly for the generation of the endocochlear potential, the driving force behind mechanoelectrical transduction. Also, fast contractile properties of outer hair cells (OHCs) allowing them to act as active signal amplifiers (Robles and Ruggero, 2001), are energetically demanding. High physiological oxidative metabolism rates in the organ of Corti may result in increased production of free radicals, more demanding to keep within a normal range. Possible convergence with different scenarios of mitochondrial dysfunction (Böttger and Schacht, 2013) makes the auditory receptor prone to imbalances in the endogenous antioxidant system, which will lead to unchecked accumulation of ROS/RNS and damage sensory cells, stria vascularis and spiral ganglion cells, leading to altered auditory function (Henderson et al., 2006; Fetoni et al., 2013; Fujimoto and Yamasoba, 2014; Alvarado et al., 2015, 2018). Meanwhile, there is also mounting evidence that the central auditory pathway is affected directly by oxidative stress leading to increases in the expression of NADPH oxidase 2 and 8-hydroxy-2-deoxyguanosine, levels of lipid peroxidation and mitochondrial DNA deletion, and also to neurodegenerative changes in the cochlear nucleus, inferior colliculus and auditory cortex (Du et al., 2014; Li et al., 2018; Tavanai et al., 2019). Supporting this, antioxidant-based therapies should mitigate the hearing damage induced by oxidative stress. Accordingly, several antioxidant and combinations has proven to be effective, mainly at the level of proof principle, for the treatment of NIHL (Yamasoba et al., 1999; Kopke et al., 2000, 2007; Tanaka et al., 2005; Le Prell et al., 2007a,^2011^), DIHL (Campbell et al., 2007; Tokgoz et al., 2011; Le Prell et al., 2014) and ARHL (Alvarado et al., 2018; Cai et al., 2020). This confirms the central role that oxidative stress plays in the development and/or progression of many auditory pathologies.

In relation to AD, the view of the disease as a proteinopathy, summarized in the amyloid cascade and tauopathy hypotheses has been the source of essential advances in the understanding of the disease, in spite of yet limited therapeutical success (Karran et al., 2011). However, this should not obscure the fact that oxidative stress has also been implicated in the pathogenesis of AD. Actually, it has even been postulated that oxidative stress-induced damage precedes AP and NFT deposits, characteristic of this neurodegenerative disease. Several biomarkers of oxidative stress have been detected in blood, neural tissue or cerebrospinal fluid in AD patients, indicating lipid peroxidation (2-propenal, 4-hydroxynonenal, F2-isoprostanes, malondialdehyde), protein oxidation (3-nitrotyrosine) and DNA oxidation (8-hydroxydeoxyguanosine, 8-hydroxyguanosine), even at early stages of the disease (Perry et al., 2002; Castellani et al., 2006; Chauhan and Chauhan, 2006; Uttara et al., 2009; Gella and Bole, 2011; Chen and Zhong, 2014; Huang et al., 2016).

Although it is not clear which factors trigger the oxidative stress in AD, both the amyloid-β protein (Aβ), either in its soluble or fibrillar forms, and the hyperphosphorylated microtubule-stabilizing protein tau, that lead to NFT formation, contribute to such oxidative stress-induced damage. Aβ has binding sites with high affinity for metal ions such as iron, copper and zinc, which would produce chelation of the Aβ and consequently accumulation of AP, with highly toxic oxidative capacity for the neurons (Chauhan and Chauhan, 2006; Uttara et al., 2009; Gella and Bole, 2011; Chen and Zhong, 2014; Huang et al., 2016). The hyperphosphorylated tau, due to the oxidative imbalance, might be modified by a non-enzymatic glycation process, with formation of advanced glycation end products (AGEs; Perry et al., 2002; Castellani et al., 2006; Chauhan and Chauhan, 2006). The AGEs are highly reactive and thus, they would generate a large amount of ROS/RNS that are not only harmful for neurons “*per se*”, but could increase, even more, Aβ levels, thus generating a vicious circle that could worsen the manifestations of AD. Nevertheless, it is also worth noting that antioxidant properties have been attributed to Aβ and hyperphosphorylated tau, suggesting that they are also part of the endogenous neuronal protection system against oxidative stress (Zou et al., 2002; Castellani et al., 2006; Baruch-Suchodolsky and Fischer, 2009; Uttara et al., 2009; Gella and Bole, 2011). Therefore, increases in ROS/RNS levels would lead directly or indirectly to an increase in the levels of those two AD biomarkers. Without ruling out other possibilities, this “dual” role seems to be related in part to the aging process, as these molecules would exert their protective effect before aging when the disease is not present yet, while their deleterious effect occurs during aging after the onset of the disease (Zou et al., 2002; Castellani et al., 2006; Baruch-Suchodolsky and Fischer, 2009; Uttara et al., 2009; Gella and Bole, 2011). As opposed to ARHL, antioxidant-based therapies have not yielded the expected results in AD. However, similar to other therapies, failures can be attributed to experimental design, including late start of the therapies when the disease is already established, the individual and not combined use of micronutrients or the difficulty of crossing the blood-brain barrier, which would decrease its bioavailability at the brain level (Persson et al., 2014). Therefore, new studies avoiding these, and other limitations are needed in order to draw valid conclusions about the use of antioxidants as possible therapies for the treatment of AD.

## Frailty Syndrome in Age-Related Hearing Loss and Alzheimer’s Disease

The World Health Organization (World Health Organization [WHO], 2017b), defines frailty as “a clinically recognizable state in which the ability of older people to cope with every day or acute stressors is compromised by an increased vulnerability brought by age-associated declines in physiological reserve and function across multiple organ systems”. Recent conceptualization currently limits consensus on how to diagnose frailty. Different models have been proposed, “the phenotype model” being one of the most used. In this model, physical/functional capacity is evaluated based on five criteria: weight loss, weakness, slowness, exhaustion, and low levels of physical activity. A patient is considered frail when presenting at least 3 and pre-frail if 1 or 2 of them are present (Xue, 2011; Wou and Conroy, 2013; Leng et al., 2014; Kane et al., 2016). It is important to note that although frailty mainly occurs during aging, frailty and aging are not synonymous and not all aged people will develop it, in fact it is estimated that, depending on diagnostic criteria, its prevalence varies between 4 to 59% among people 65 years or older (Kojima et al., 2019). Although frailty is a multifactorial condition that affects multiple organs and systems, underlying mechanisms are not clear. It has been proposed that pro-inflammatory states, sarcopenia, hormonal and metabolic imbalances, DNA damage, and more recently, oxidative stress are critical in the development of this pathology (Mulero et al., 2011; Xue, 2011; Inglés et al., 2014; Leng et al., 2014; Namioka et al., 2017; Soysal et al., 2017; World Health Organization [WHO], 2017b; Bisset and Howlett, 2019; El Assar et al., 2020). Regarding the latter, it has been suggested that it could be a primary mechanism triggering frailty, since oxidative stress-induced damage could serve as background or starting point of the multiple alterations described in subjects with frailty (Mulero et al., 2011; El Assar et al., 2020). For instance, while the oxidative stress-induced mitochondrial dysfunction could contribute to the distinctive sarcopenia that leads to weight loss, weakness and slowness, the DNA oxidation would induce the DNA damage observed in frailty (El Assar et al., 2020).

**FIGURE 1:**
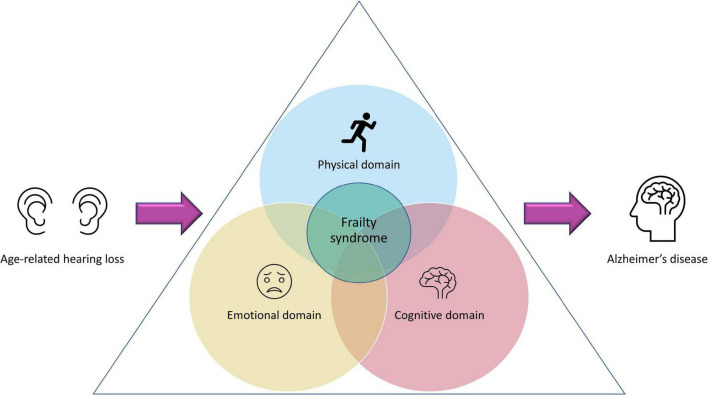
Age-related hearing loss and frailty syndrome. Presbycusis has been proposed as a preventable risk factor for the development of frailty. Depending on the magnitude of the hearing loss, its impact could go far beyond auditory dysfunction, affecting also the physical, emotional, and cognitive domains. If presbycusis-induced alterations of these three domains co-exist, this could lead to a “frailty syndrome” that, consequently, will have a profound negative impact in the aggravation of Alzheimer’s disease, if there is comorbidity between FS and AD.

Given the relevance that, from the clinical point of view, frailty has for different age-related pathologies, the concept has evolved, incorporating in addition to the physical/functional domain, the emotional and cognitive, known as frailty syndrome (FS; Xue, 2011; Wou and Conroy, 2013; Leng et al., 2014; Khezrian et al., 2017; Kojima et al., 2019). It has been suggested that the FS, mainly the emotional and cognitive domains, are essential for the development or progression of AD, which has a high prevalence (32%) of coexisting frailty (Kojima et al., 2019). This is crucial considering that the potentially modifiable nature of the risk factors that could lead to emotional or cognitive frailty, might be useful to identify possible therapeutic targets for AD. For instance, it has been proposed that cognitive frailty is pivotal in the pathogenesis of AD. One of the key risk factors for the development of this frailty is hearing impairment and particularly, ARHL (Figure 1) (Panza et al., 2006, 2015a,b, 2019; Khezrian et al., 2017). As previously mentioned, epidemiological studies highlight the association between hearing loss in older adults with dementia and cognitive decline. In these subjects, the rate of cognitive decline is accelerated, increasing the risk of developing dementia, including AD, and this risk is proportional to the level of hearing loss observed (Lin et al., 2011; Lin and Albert, 2014; Deal et al., 2016; Thomson et al., 2017). The emergence of the concept of FS, opens the possibility that at least part of the high prevalence of hearing loss observed in patients with AD and its consequences for the progression of the disease may be amplified through FS, and this will need to be explored.

Regarding the emotional frailty, ARHL has a profound impact on the quality of life of these patients causing low self-esteem, social withdrawal, isolation, frustration, and depression which would undoubtedly affect their emotional domain (World Health Organization [WHO], 2002, 2017a,b; Huang and Tang, 2010; Ciorba et al., 2012; Kidd and Bao, 2012), producing or aggravating the emotional frailty (Figure 1). Since emotional frailty can coexist with cognitive frailty (Khezrian et al., 2017), the latter can be affected, which would increase the risk of suffering from dementia and AD in older people. Finally, the relationship between ARHL and physical frailty has been less studied. However, there is no doubt that hearing loss in older people also has a negative impact on their physical activity. Accordingly, it has been suggested that ARHL: (1) induces to perform less physical activity because subjects are socially isolated; (2) affects cognitive resources and attention, which are essential for maintaining posture and balance and (3) restricts the ability to effectively monitor the environment (hearing footsteps and other auditory cues that provide guidance), which reduces the probability of engaging in physical activities (Gispen et al., 2014; Fortunato et al., 2016). Additionally, common neural degeneration, which affects both the cochlea and the vestibular organ, involved in controlling balance, may explain decreases in physical activity (Gispen et al., 2014; Fortunato et al., 2016). All these scenarios may influence the onset and/or progression of physical frailty (Figure 1). Finally, a high association between presbycusis and falls over time in older people has been described, which might have repercussions leading to decreased physical activity and therefore, worsening frailty (Kamil et al., 2016).

## Conclusion

**FIGURE 2:**
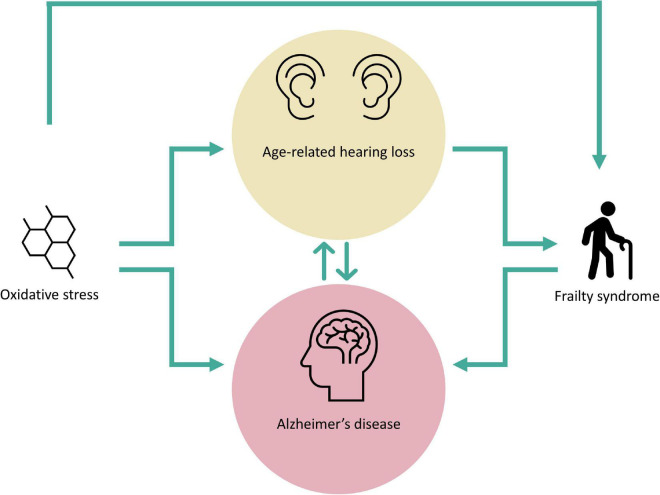
Oxidative stress and frailty syndrome in age-related hearing loss and Alzheimer’s disease. The figure shows the complex interplay among oxidative stress, frailty syndrome, age-related hearing loss and Alzheimer’s disease. Oxidative stress-damage represents a possible trigger for both age-related hearing loss and Alzheimer’s disease, and also for the genesis of frailty syndrome. Presbycusis is a fundamental risk factor for the development of Alzheimer’s disease and frailty syndrome while, frailty syndrome may contribute to the exacerbation of Alzheimer’s disease. Note a hypothetical biunivocal relationship between age-related hearing loss and Alzheimer’s disease, as these neurodegenerative pathological conditions may influence each other.

Oxidative imbalance and related FS due to it, may be part of multiple pathologies that affect geriatric patients. The oxidative imbalance, either due to overproduction or lack of elimination of ROS is critical not only for the onset and/or development of ARHL or AD but also for triggering FS (Figure 2). The latter is also affected by ARHL which in turn, enhances the frailty condition in older subjects. Considering that oxidative stress, ARHL and FS individually might be critical factors in the development of AD (Figure 2), their coexistence in the same patient would be decisive for the onset, progression, and severity of this neurodegenerative disease. More so if it is considered that dementias such as AD may negatively affect ARHL, generating a vicious circle between both conditions with devastating consequences. Hence, due to the complex relationship that exists between ARHL and AD in which oxidative stress and related FS may play unexplored roles, it would be logical to expect that therapies aimed at specific targets of a single pathogenic pathway would have little beneficial effect. Therefore, the optimal therapeutic approach should not only focus on a single pathway, instead it should be multifaceted, as the combination of therapeutic strategies aimed at different pathogenic pathways or different points of the same pathway would guarantee a synergistic interaction enhancing the beneficial effect of the therapy which in turn, would have a positive impact on the patient’s health quality of life.

To achieve the goal of unveiling pathogenic mechanisms of AD in relation to ARHL, it is necessary to design studies in appropriate animal models reproducing the pathological complexities of AD, in particular comorbidity with ARHL, oxidative stress and frailty syndrome. Such animal models, besides presenting clinical and biological markers of AD, must reproduce ARHL traits, comparable to those found in humans. The results obtained from such studies will allow a more “humanized” and reality-adjusted analysis of the data that will help to comprehend the interplay between these important neurodegenerative pathologies, which will facilitate a better understanding of the etiopathogenic mechanisms involved in their onset, progression, and severity.

## Author Contributions

JA and VF-S: drafting of the manuscript and design of figures. JA, VF-S, and JJ: critical revision of the manuscript for important intellectual content. All authors contributed to the article and approved the submitted version.

## Conflict of Interest

The authors declare that the research was conducted in the absence of any commercial or financial relationships that could be construed as a potential conflict of interest.

## Publisher’s Note

All claims expressed in this article are solely those of the authors and do not necessarily represent those of their affiliated organizations, or those of the publisher, the editors and the reviewers. Any product that may be evaluated in this article, or claim that may be made by its manufacturer, is not guaranteed or endorsed by the publisher.
